# Persistent 18F-FDG Brain PET Fronto-Temporal Hypometabolism and Cognitive Symptoms Two Years after SARS-CoV-2 Infection: A Case Report

**DOI:** 10.3390/neurolint15030058

**Published:** 2023-07-25

**Authors:** Stefania Rossi, Elena Prodi, Rosalba Morese, Gaetano Paone, Teresa Ruberto, Leonardo Sacco

**Affiliations:** 1 Department of Neurology, Neuropsychology and Speech Therapy Unit, Neurocenter of Southern Switzerland, Ente Ospedaliero Cantonale, 6900 Lugano, Switzerland; leonardo.sacco@eoc.ch; 2 Department of Neuroradiology, Neurocenter of Southern Switzerland, Ente Ospedaliero Cantonale, 6900 Lugano, Switzerland; elena.prodi@eoc.ch; 3 Faculty of Biomedical Sciences, Università della Svizzera Italiana, 6900 Lugano, Switzerland; rosalba.morese@usi.ch (R.M.); gaetano.paone@eoc.ch (G.P.); 4 Faculty of Communication, Culture and Society, Università della Svizzera Italiana, 6900 Lugano, Switzerland; 5 Department of Nuclear Medicine, Imaging Institute of Southern Switzerland, Ente Ospedaliero Cantonale, 6500 Bellinzona, Switzerland; teresa.ruberto-macchi@eoc.ch

**Keywords:** SARS-CoV-2, post-COVID-19, cognitive impairment, Alzheimer’s disease, FDG-PET

## Abstract

At least 10% of patients experience persistent symptoms after SARS-CoV-2 infection, a condition referred to as post-acute COVID-19, post-acute sequelae of SARS-CoV-2 infection (PASC), long COVID, long-haul COVID, long-term effects of COVID, post-COVID-19 and chronic COVID. In this report, we describe a case of persistent cognitive deficits developed after SARS-CoV-2 infection in a 40-year-old woman with a family history of early-onset Alzheimer’s disease (EOAD) since her father was diagnosed with EOAD at the age of 50. We describe the clinical picture and workup, with special emphasis on the alterations of brain glucose metabolism evidenced by 18-fluoro-deoxy-glucose positron emission tomography (FDG-PET), which could be considered a useful marker of the presence and persistence of cognitive deficits.

## 1. Introduction

An acute SARS-CoV-2 infection typically resolves in 2 to 6 weeks. However, at least 10% of COVID-19 survivors, which is likely underestimated, can have symptoms that last up to several months after recovery from an acute infection [1]. The World Health Organization (WHO) has defined post-COVID-19 as a ‘condition that occurs in individuals with a history of probable or confirmed SARS-CoV-2 infection, usually 3 months from the onset, with symptoms that last for at least 2 months and cannot be explained by an alternative diagnosis’. Common symptoms include, but are not limited to, fatigue, shortness of breath and cognitive dysfunction, which generally have an impact on everyday functioning. Symptoms might have a new onset following initial recovery from an acute COVID-19 episode or persist from the initial illness. Symptoms might also fluctuate or relapse over time [2] (p. e102). The term ‘long-COVID’ was first coined and disseminated through social media by patients experiencing persistent symptoms and/or delayed or long-term complications beyond the acute infection. Subsequently, kindred terms were introduced, including post-acute COVID-19, post-acute sequelae of SARS-CoV-2 infection (PASC), long COVID, long-haul COVID, long-term effects of COVID, post-COVID-19 and chronic COVID [3].

Post-COVID-19 development appears to be dependent on pre-existing risk factors and the severity of the acute infection. The main risk factors appear to be female gender, the presence of more than five early symptoms, and the initial acute severity of COVID-19 [1]. However, anyone exposed to SARS-CoV-2 can experience persistent or late symptoms, even people who had a mild illness and were not hospitalized. Symptoms improve over time in most cases, but there is variability in the time taken for symptom resolution. To date, the impact of new SARS-CoV-2 variants and the role of vaccination on the onset of post-COVID-19 need to be clarified [4,5].

The pathological mechanisms behind post-COVID-19 infection are still unclear but may include direct central nervous system infection, neuroinflammatory effects of distal inflammation, autoimmunity, reactivation of latent herpes virus infection, neurovascular disease and hypoxia [6]. The most supported theory is an autoimmune process with an excessive innate immune response and activation of cytokines [7]. Some aspects of this condition are unique to SARS-CoV-2, but many appear to be similar to recovery from other post-viral illnesses (Lyme disease, Epstein-Barr virus, Cytomegalovirus and Zika) or other conditions such as myalgic encephalomyelitis/chronic fatigue syndrome (ME/CFS), fibromyalgia and postural orthostatic tachycardia syndrome (POTS).

Diverse neurological symptoms are described in post-COVID-19, including sensorimotor symptoms, memory loss, cognitive impairment, dizziness, sensitivity to light and noise, loss of smell or taste and autonomic dysfunction. Increases in anxiety, depression and sleep disorders are also reported [8,9].

Fatigue and cognitive impairment are among the most common and debilitating symptoms of post-COVID-19 syndrome. They are reported to occur, respectively, in 32% and 22% of individuals, 12 or more weeks after acute SARS-CoV-2 infection, as outlined by a recent meta-analysis by Ceban et al. [8]. Post-COVID-19 fatigue is defined as a ‘decrease in physical and/or mental performance resulting from changes in central, psychological and/or peripheral factors resulting from COVID-19 disease’ [10] (p. 1). Among cognitive deficits, attentive-executive functions and episodic memory are the most affected domains, as reported by a recent review by Tavares et al. [11]. Headaches are also a common feature of post-COVID-19. It can manifest either as a tension-type-like or a migrainous phenotype [12].

Post-COVID-19 neurologic symptoms are diverse and overlap with other neurologic conditions. To date, no diagnostic biomarker is available, and there is no specific treatment. Different reports demonstrated brain fluorine-18 fluoro-deoxy-glucose positron emission tomography/computed tomography (18F-FDG PET/CT) hypometabolism in various brain regions in post-COVID-19 patients, such as the orbitofrontal cortex, including the olfactory gyrus, the right temporal lobe, the amygdala, hippocampus, the right thalamus, pons, medulla, bilateral cerebellum, fusiform gyri and in the left insula [10]. The 18F-FDG PET/CT could represent a potential tool to outline brain changes in post-COVID-19.

## 2. Case Description

A 40-year-old healthy female, who is right-handed, with a master’s degree, employed as a manager in a nursing home for the elderly, with no significant medical history, underwent SARS-CoV-2 infection in December 2020. She was not vaccinated for COVID-19 at the time of infection. Acute symptoms included headache, fatigue, loss of sense of smell and taste, shortness of breath and mild fever. She did not require hospitalization. Cognitive symptoms, including brain fog, difficulty concentrating, forgetfulness and word retrieval problems, were quickly noticed by the patient when she returned to work after 10 days. She also experienced sleep problems, characterized by disrupted sleep, and hair loss. Because the symptoms persisted 10 months after COVID infection, she was evaluated at a dedicated COVID-19 clinic, where she underwent a comprehensive physical, cognitive and neurological assessment. Persistent symptoms included olfactory and gustatory disorders, tension headache, sleep disturbances, cognitive deficits and fatigue. She did not complain of physical symptoms such as dyspnea, chest pain or a cough and she denied psychological compliant such as anxiety or depression. Neurologic examination, including assessment of cranial nerves function, reflexes, motor function, sensation, gait and coordination, did not reveal abnormal results. Routine laboratory tests, including thyroid function evaluation, ferritin, folate, vitamin B12 and antibodies against Borrelia, Treponema Pallidum and HIV, showed normal values except for a vitamin D deficiency. She scored 28/30 on the Montreal Cognitive Assessment (MoCA) [13]. Detailed neuropsychological testing revealed clinically relevant fatigue on the Chalder Fatigue Scale [14] and a deficit in a cancellation test developed to measure concentration and attention (D2 Sustained-Attention Test) [15]. Other cognitive domains appeared normal (Table 1, Visit 1).

**Figure 1:**
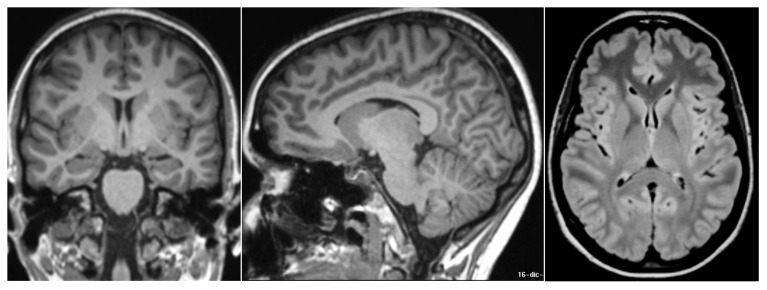
Normal findings at brain magnetic resonance imaging (MRI). Coronal and sagittal T1-weighted and axial FLAIR images show absence of mesial temporal lobe and cortical of atrophy and absence of signal changes.

She underwent brain magnetic resonance imaging (MRI) with contrast in a 3 Tesla scanner, including volumetric T1-weighted sequences to score for brain atrophy, which was unremarkable (Figure 1).

At this time, sleep disorders were treated with prolonged-release melatonin and magnesium, and the vitamin D deficiency was treated with a high-dose vitamin D supplement.

Despite following the vaccination program, the patient reported a second SARS-CoV-2 infection in December 2021, with minor symptoms. At a follow-up neurological evaluation, about 18 months after acute COVID infection, the patient reported a restored sleep pattern but persistent fatigue and attention deficits, interfering with job activities and overall quality of life. The neurological evaluation was unremarkable. The EEG examination was normal. However, neuropsychological assessment, using parallel versions of previous tests, showed a slightly decreased total MoCA score (26/30) and mild cognitive impairment in a word list learning test (Rey auditory verbal learning test, AVLT), a widely used task for assessing verbal episodic memory (learning, storage, recall and recognition). (Table 1, Visit 2) [16]. Again, she did not show psychological and psychiatric symptoms, obtaining normal scores in the Hospital Anxiety and Depression Scale (HADS) [17], a test developed to measure anxiety and depression in a general medical population. Based on the subtle clinical worsening and considering the family history of early-onset Alzheimer’s Disease (EOAD), the patient was referred to our Memory Clinic for a comprehensive evaluation. In fact, the patient’s father was diagnosed with EOAD at the age of 50. The diagnosis was supported by brain MRI findings of bilateral temporo-parietal atrophy, 18F-FDG PET hypometabolism in the temporal lobe and posterior parietal lobes and low beta-amyloid and increased total tau, and hyperphosphorylated tau in cerebrospinal fluid (CSF).

At the time of the visit to our Memory Clinic, the patient reported an increase in headache symptoms. The pain was not associated with nausea, photophobia, phonophobia or autonomic symptoms. She underwent treatment with Amitriptyline 10 milligrams (mg) a day, with no benefit, followed by Topiramate 30 mg a day. A neuropsychological assessment (Table 1, Visit 3) revealed poor performance in the long verbal memory task, with a deterioration in the score compared to the previous evaluation. The burden of fatigue, anxiety and depression symptoms did not increase in the self-reporting questionnaires. Nonetheless, we noticed an increase in psychophysical burden as a stressful reaction to post-COVID symptoms. We proposed a psychotherapeutic approach to the patient.

**Figure 2:**
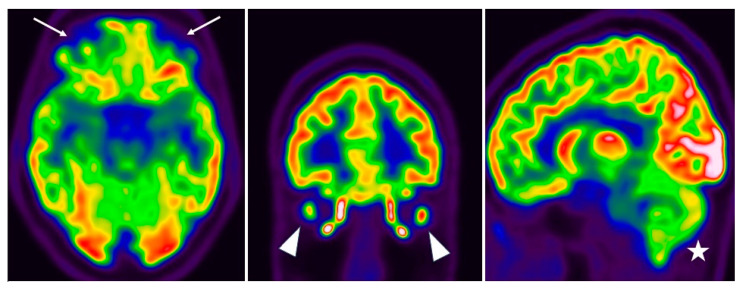
The 18F-FDG PET on axial, coronal and sagittal planes showed bilateral hypometabolism in the inferior frontal lobe (olfactory gyrus) (arrow), in the temporal lobe (limbic/paralimbic regions) (arrowhead) and in the cerebellum (star).

**Figure 3:**
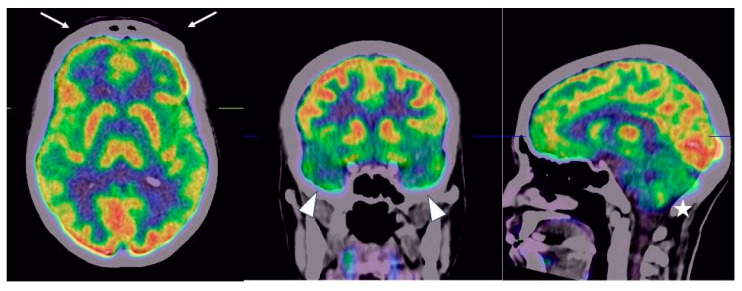
Fused 18F-FDG PET/CT images on axial, coronal and sagittal planes confirmed the reduction of tracer distribution in the aforementioned cerebral regions, inferior frontal lobe (olfactory gyrus) (arrow), in the temporal lobe (limbic/paralimbic regions) (arrowhead) and in the cerebellum (star). On the co-registered CT image, no-specific alterations of cerebral parenchyma were observed.

The patient underwent a brain flourine-18 fluoro-deoxy-glucose positron emission tomography/computed tomography (18F-FDG PET/CT) in July 2022, that demonstrated a hypometabolic profile in the inferior frontal lobe (olfactory gyrus), in the temporal lobe (limbic/paralimbic regions) and in the cerebellum (Figure 2 and Figure 3).

We also performed a CSF composition study and sent a CSF sample to an outside laboratory to examine for a variety of neurodegenerative indicators and paraneoplastic antibodies in order to investigate further causes of cognitive impairment. (Table 2). CSF parameters were normal and did not support a diagnosis of biomarker-confirmed Alzheimer’s disease, based on the latest diagnostic criteria [18]. Notably, a low Ab42/40 ratio provides more robust evidence of amyloid negativity and provides a better predictive value for the conversion to Alzheimer’s disease [19,20]. The diagnosis of paraneoplastic syndrome was also ruled out [21].

An Alzheimer’s disease genetic test was not carried out as per the patient’s and her father’s preferences.

**Figure 4:**
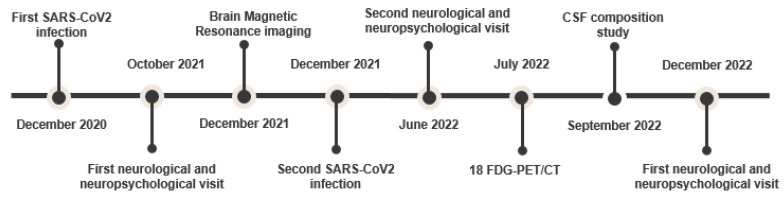
Timepoints of medical history, clinical assessment and diagnostic tests.

Figure 4 summarizes the timepoints of the medical history, clinical assessment and diagnostic tests.

## 3. Discussion

We describe a 40-year-old patient with cognitive deficits including memory impairment, inattentive symptoms, fatigue, sleep disturbance and headaches, which developed after SARS-CoV-2 infection. These symptoms still persist more than 24 months after acute infection. The 18F-FDG PET/CT performed 18 months after the acute infection showed areas of brain hypometabolism. Diagnostic investigations have excluded other causes of rapidly progressive dementia or Alzheimer’s disease, a particular concern due to the family history of early-onset Alzheimer’s disease (EOAD). EOAD has different features compared to late-onset Alzheimer disease (LOAD). In EOAD the amnesic presentation is less common and different presentations including posterior cortical atrophy, frontal variants and language deficits (primary progressive aphasia) are recognized [22,23]. The most thoroughly described EOAD phenotype is posterior cortical atrophy (PCA), which is characterized by difficulties in space and object perception. Patients with the frontal variant of Alzheimer’s disease show impairment in executive function and behavior. The logopenic form of primary progressive aphasia typically manifests as a non-fluent aphasia with prominent word-finding pauses, naming and repetition difficulties [24,25].

The absence of CSF pleocytosis or oligoclonal bands allowed us to rule out active inflammation. A wide spectrum of paraneoplastic antibodies in CSF was investigated to exclude a paraneoplastic syndrome. Causes of secondary headaches were ruled out and a depressive status that could trigger headaches was absent. Although we did not perform a genetic analysis for Alzheimer’s disease, the atypical neuropsychological pattern, 18F-FDG PET/CT and CSF findings, together with the onset of symptoms related to SARS-CoV-2 infection, argued against this hypothesis. Notably, biomarkers such as elevated CSF tau (both total tau and phosphorylated tau) and low levels of CSF amyloid-β1-42 (Aβ42) have been incorporated in the diagnostic criteria for Alzheimer disease [18,24]. Overall, symptoms were interpreted as post-COVID-19 syndrome.

Regarding neuropsychological assessments, the RAVLT showed a progressive decline in delayed recall, with maintained overall delayed recognition performance; this is different from the typical Alzheimer’s disease encoding and storage deficit. Early-onset Alzheimer’s disease is commonly characterized by smaller memory deficits and a greater impairment of other functions, including language, visuospatial, executive, and motor functions and behavioral dysregulation [22]. The global cognitive profile of our patient was preserved, except for episodic memory deficit and low processing speed. Neuropsychological findings showed a pattern of word retrieval characterized by preserved primacy/recency effect [25], meaning that information presented at the beginning (primacy) and end (recency) of a learning test tends to be retained better than information presented in the middle. The evidence shows that the primacy/recency effect results from processing of two different memory systems, the long-term and short-term memory [26]. The verbal episodic memory anomalies in our patient could be related to an ineffective use of strategies to store information rather than being related to a pure memory deficit. A selective mild executive dysfunction could interfere with material organization and successful recall of information presented in the middle of the task [27]. Those data confirm a neuropsychological pattern different from Alzheimer’s disease [27] and amnestic mild cognitive impairment [24]; both presented accelerated memory decay for the early and middle part of a list of words, in line with a genuine episodic memory deficit and often expression of the same biological disease at different stages.

Episodic memory has already been tested in COVID patients using the AVLT test, as we did, early after the acute infection, with evidence of memory impairment, especially in free and delayed verbal recall and recognition. However, in this study, the primacy/recency effect and episodic memory performances long after the acute infection have not been tested [28].

An 18F-FDG PET/CT, performed 18 months after the acute infection, demonstrated hypometabolism in fronto-orbital regions including the olfactory gyrus, the limbic and paralimbic areas and the cerebellum. This pattern of hypometabolism in post-COVID-19 has been shown by other authors [29,30,31,32]. Guedj et al. demonstrated, in adult patients with post-COVID-19 (26–155 day after acute infection), hypometabolism in the bilateral rectal/orbital gyrus, including the olfactory gyrus, in the right temporal lobe, in the amygdala and the hippocampus, extending to the right thalamus, pons, medulla and cerebellum [32]. A similar FDG-PET pattern has been shown in a pediatric post-COVID-19 series, evaluated on average 5 months (range 1–8 months) after acute infection, with hypometabolism in the bilateral medial temporal lobes, brainstem and cerebellum and also the right olfactory gyrus [33]. Rudroff and colleagues, in a review about 18F-FDG PET findings in post-COVID-19 patients, outlined that most studies demonstrated hypometabolism in the frontal lobes or cortical-subcortical networks of frontal hubs [10]. An inflammatory process targeting the frontal lobes and frontal network could be the underlying cause of these abnormalities in post-COVID-19 [34]. This cortical metabolic anomaly pattern may overlap with the one found in the frontal variant of Alzheimer’s disease. On the contrary, 18F-FDG PET hypometabolism in late-onset Alzheimer’s disease typically involves the posterior cingulate, the temporo-parietal and the prefrontal association cortex [35].

Notably, there are studies that did not report brain 18F-FDG PET abnormalities in patients complaining of neurocognitive symptoms more than 3 months after acute SARS-CoV-2 infection [36,37].

Also, in rare cases of post COVID-19, a diffuse brain 18F-FDG PET hypometabolism has been described, requiring differential diagnosis with Alzheimer’s disease. To date, only one case in the literature describes the use of dual imaging with FDG PET and 18F-amyloid PET/CT to assess the presence of Aβ plaques [38]. Further studies are needed to understand the links between inflammation and neurodegeneration in post COVID-19.

The peculiarity of this case is to observe metabolic changes 18 months after the acute infection and to study the details of the 18F-FDG-PET and neuropsychological examination with respect to a possible Alzheimer’s profile. Different time ranges of brain 18F-FDG PET evaluations in post-COVID-19 patients are described in the literature. To the best of our knowledge, this appears to be the first case to highlight functional sequelae after such a long period from COVID-19 infection. Long-term follow-ups will allow us to assess the evolution of these abnormalities and associated clinical symptoms.

## 4. Conclusions

This report outlines how FDG-PET may represent a tool to identify brain involvement in long COVID-19. Still, the significance of brain FDG PET metabolic abnormalities remains to be determined. Moreover, the duration and reversibility of these metabolic abnormalities remain to be elucidated.

## Figures and Tables

**Table 1:** Neuropsychological tests panel.

Task	Visit 1	Visit 2	Visit 3
MoCA	28/30	26/30	28/30
Digit Span forward	6/9	6/9	5/9
Digit Span backward	5/9	5/9	5/9
Rey auditory verbal learning test (AVLT): Immediate total recall	43/75	40/75 *	37/75 *
Rey auditory verbal learning test (AVLT): Delayed total recall	11/15	8/15 *	5/15 *
Rey auditory verbal learning test (AVLT): Delayed recognition	14/15	10/15	10/15
D2 Sustained-Attention Test: speed	130 *	133 *	119 *
D2 Sustained-Attention Test: error rate (%)	5.2	3.07	3.3
D2 Sustained-Attention Test: performance	126	126	114
Modified Five Point Test: unique designs	34	55.8	--
Modified Five Point Test: strategy score	40	67.5	--
Modified Five Point Test: percentage of perseverations	2.94	2.5	
Phonemic Fluency	36	37	
Chalder Fatigue Scale (CFS-11)	8/11 *	--	1/11
Hospital Anxiety and Depression scale (HADS): Anxiety	--	3/21	4/21
Hospital Anxiety and Depression scale (HADS): Depression	--	2/21	4/21

Performances on neuropsychological tests are presented as raw scores. The visits were performed by different operators. * Indicates pathological performance, defined by a score of two standard deviations below the normative means, corrected according to the patient’s age and education level (Equivalent Scores, ES). ES is a 5-point scale that offers a solution to the problem of standardizing neuropsychological scores after adjustment for age and education. ES = 0 reflects a pathological performance. ES = 1 a borderline performance. ES ≥ 2 a normal performance.

**Table 2:** CSF panel.

Parameters	Results
Appearance	Clear
Protein	321 mg/L
Leukocytes	0.7 Lc/μL
Albumin	205 mg/L
Oligoclonal bands	0
Tau protein (normal reference < 452 ng/L)	201 ng/L
P Tau protein (normal reference < 61 ng/L)	33 ng/L
Aß42 (normal reference > 630 ng/L)	1730 ng/L
Aß40	8239
Ratio Aß42/40 (ref. > 0.1)	0.21
Anti-NMDAR, IgG	Negative
Anti-CASPR2, IgG	Negative
Anti-LGI1, IgG	Negative
Anti-DPPX	Negative
Anti-AMPA-R	Negative
Anti-IgLON5R	Negative
Anti-mGluR5	Negative
Anti-GlyR	Negative
	Negative

SF, cerebrospinal fluid; P Tau protein, phosphorylated tau protein; Aß42, ß-amyloid 42; Aß40, ß-amyloid 40; ratio Aß42/40, ratio ß-amyloid 42/ß-amyloid 40; NMDAR, N-methyl-d-aspartate; CASPR2, contactin-associated protein-like 2; LGI1: leucine-rich glioma inactivated 1; DPPX, Dipeptidyl-peptidase-like protein 6; GABABR: γ-aminobutyric acid type B receptor; AMPAR, α-amino-3-hydroxy-5-methyl-4-isoxazolepropionic receptor; IgLON5R, immunoglobulin-like cell adhesion molecule 5; mGluR5, metabotropic glutamate receptor 5; GlyR, glycine receptor. The laboratory’s internal cut-offs have been used.

## Data Availability

Not applicable.
